# Comparison of P2X and TRPV1 receptors in ganglia or primary culture of trigeminal neurons and their modulation by NGF or serotonin

**DOI:** 10.1186/1744-8069-2-11

**Published:** 2006-03-28

**Authors:** Manuela Simonetti, Alessandra Fabbro, Marianna D'Arco, Marina Zweyer, Andrea Nistri, Rashid Giniatullin, Elsa Fabbretti

**Affiliations:** 1 Neurobiology Sector, International School for Advanced Studies (SISSA), Via Beirut 4, 34014 Trieste, Italy; 2 Department of Normal Human Morphology, University of Trieste, Via Manzoni 16, 34138 Trieste, Italy

## Abstract

**Background:**

Cultured sensory neurons are a common experimental model to elucidate the molecular mechanisms of pain transduction typically involving activation of ATP-sensitive P2X or capsaicin-sensitive TRPV1 receptors. This applies also to trigeminal ganglion neurons that convey pain inputs from head tissues. Little is, however, known about the plasticity of these receptors on trigeminal neurons in culture, grown without adding the neurotrophin NGF which *per se *is a powerful algogen. The characteristics of such receptors after short-term culture were compared with those of ganglia. Furthermore, their modulation by chronically-applied serotonin or NGF was investigated.

**Results:**

Rat or mouse neurons in culture mainly belonged to small and medium diameter neurons as observed in sections of trigeminal ganglia. Real time RT-PCR, Western blot analysis and immunocytochemistry showed upregulation of P2X_3 _and TRPV1 receptors after 1–4 days in culture (together with their more frequent co-localization), while P2X_2 _ones were unchanged. TRPV1 immunoreactivity was, however, lower in mouse ganglia and cultures. Intracellular Ca^2+ ^imaging and whole-cell patch clamping showed functional P2X and TRPV1 receptors. Neurons exhibited a range of responses to the P2X agonist α, β-methylene-adenosine-5'-triphosphate indicating the presence of homomeric P2X_3 _receptors (selectively antagonized by A-317491) and heteromeric P2X_2/3 _receptors. The latter were observed in 16 % mouse neurons only. Despite upregulation of receptors in culture, neurons retained the potential for further enhancement of P2X_3 _receptors by 24 h NGF treatment. At this time point TRPV1 receptors had lost the facilitation observed after acute NGF application. Conversely, chronically-applied serotonin selectively upregulated TRPV1 receptors rather than P2X_3 _receptors.

**Conclusion:**

Comparing ganglia and cultures offered the advantage of understanding early adaptive changes of nociception-transducing receptors of trigeminal neurons. Culturing did not prevent differential receptor upregulation by algogenic substances like NGF or serotonin, indicating that chronic application led to distinct plastic changes in the molecular mechanisms mediating pain on trigeminal nociceptors.

## Background

Trigeminal ganglion (TG) neurons convey sensory inputs including painful stimuli from head tissues like skin and mucosal surfaces, tooth pulp and meninges. The characterization of nociception-transducing receptors on TG neurons thus becomes important to understand certain forms of acute and chronic pain.

Important pain transducers of noxious stimuli are small and medium size neurons (nociceptors) that can express ATP-activated P2X_3 _subunit-containing receptors and/or capsaicin (and heat) sensitive TRPV1 receptors [1,2]. Activation of TRPV1 receptors is associated with a slow inward current [1] while ionotropic ATP receptors generate fast currents mediated by P2X_3 _receptors, and slow ones mediated by P2X_2 _subunit-containing receptors [3,4]. Over-expression of heteromeric P2X_2/3 _receptors is suggested to be associated with chronic pain states [2,5].

To understand the molecular basis of chronic pain, it would be helpful to use TG neurons in culture as models to study slow changes in the structure and function of P2X or TRPV1 receptors after exposure to mediators such as serotonin or NGF to mimic certain forms of chronic headache [6].

TG nociceptive neurons are modulated by serotonin (5-HT) in a complex fashion. In fact, 5-HT can excite them through 5-HT_3 _receptors [7] as well as depress their pain signaling via multiple subtypes of the 5-HT_1 _receptor group [8], an action which had led to the clinical use of 5-HT_1 _receptor agonists to treat migraine. Furthermore, acute application of 5-HT can strongly potentiate responses mediated by TRPV1 receptors, indicating rapid nociceptive sensitization [9]. Nevertheless, headache is usually a sustained form of pain and its molecular mechanisms including the modulatory action of 5-HT on pain signaling by TG neurons should be studied with long-term experimental models.

NGF may be an additional contributor to headache because of its increased levels in the cerebrospinal fluid of patients during headache attacks [10]. Application of NGF sensitizes spike firing and TRPV1 receptor activity of dorsal root ganglion (DRG) neurons [1,43] and facilitates release of algogenic substances like CGRP from TG neurons [11].

To the best of our knowledge, there is no information on the evolution of TG pain receptors (ATP P2X or TRPV1 ones) during culture since previous studies have simply investigated nociceptors *after *they had been plated for culture [4,6,12]. Thus, the current study characterized the expression, distribution and function of ATP P2X and TRPV1 receptors in cultured trigeminal neurons in comparison with ganglia. We chose to study rat and mouse neurons because the former had been used in other studies of pain and the latter can provide fundamental new data concerning genetic models of chronic pain. While information concerning P2X receptors in TG is less abundant than those for DRG, it is clear that extrapolating data from DRG to TG is inadvisable in view of the very different distribution, expression and modulation of P2X_3 _receptors between these ganglia [13].

Using TG preparations, we addressed the following questions: 1. How do P2X and TRPV1 receptors of rat or mouse TG neurons grown in culture compare with those of ganglia? 2. Are these markers stable in culture and are they functional? 3. Are these pain receptors similar in rat and mouse TGs? 4. Can 5-HT or NGF modulate the function of P2X and TRPV1 receptors on cultured TG neurons? We report significant differences in the expression and pharmacological modulation of P2X and TRPV1 receptors of nociceptive neurons cultured from rat or mouse TGs.

## Results

### Rat and mouse neurons are stable in culture without adding NGF

Scanning electron microscopy was used to investigate if the surface ultrastructure of rat or mouse TG neurons had changed after 24 h in culture. After enzymatic dissection of the ganglion at one d in culture, rat or mouse TG neurons retained their spherical shape with somewhat irregular contour probably resulting from the detachment of satellite cells. The surface of TG neurons in culture was characterized by the presence of small microvilli of different length (200–500 nm) emerging from cell body as well as by filamentous structures (2–10 μm long, with a cross-section of 120 nm) that remained in contact with the neuron surface for their entire length. These morphological features of TG neurons are typical of rat sensory neurons [14], confirming that culturing conditions without exogenous NGF did not change neuronal phenotype.

**Figure 1:**
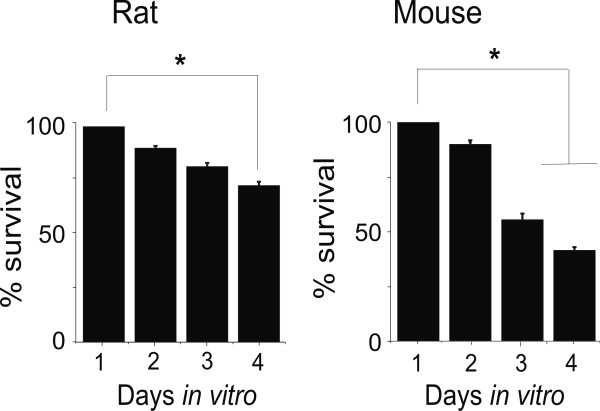
**Survival of neurons in TG ganglia or in culture**. Survival of neurons (calculated as neuron density expressed as number of β-tubulinIII positive cells per unit area) at 1–4 days in culture. Data are normalized with respect to those at 1 day (n = 250). *: P < 0.05.

Rat β-tubulinIII positive cells demonstrated good viability *in vitro *(<30 % loss at 4 d; Fig. 1, left, in which data expressed as neuronal density per unit area are normalized with respect to the number of neurons found at 24 h in culture). Mouse neurons were more labile as their number was more consistently reduced after the second d in culture (Fig. 1, right). Nevertheless, at each one of the four days in culture, only 3–10 % of total mouse neurons was labeled with antibodies against early markers of apoptosis as JNK or activated caspase3 (not shown). Apoptotic nuclei were also rarely detected.

### mRNA and protein expression

**Figure 2:**
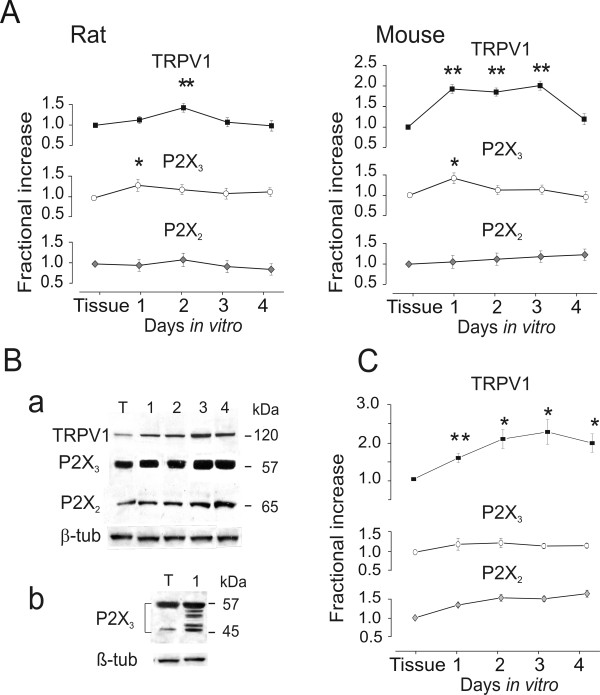
**Real time RT-PCR and western immunoblot of ganglia and cultures**. A, real time RT-PCR of ganglia and cultures from rat and mouse. Ordinate: relative increment with respect to the ganglion products. For each receptor, amplification values were normalized with β-tubulinIII mRNA levels and compared with the ganglion mRNA levels. GAPDH amplification control was the same in all reactions. B, western immunoblots of equal amounts of neuronal protein lysates (β-tubulinIII) derived from ganglia or culture. Immunodetection of P2X_3_, P2X_2 _and TRPV1 mature proteins revealed proper migration (a) in accordance with Vulchanova *et al*.[53] for the predominant P2X_3 _form (57 kDa), with Newbolt *et al*. [54] for the P2X_2 _mature receptor (65 kDa) and with Kedei *et al*. [55] for the TRPV1 mature receptor (120 kDa). Panel B b shows P2X_3 _native (45 kDa) and intermediate polypeptides (up to 50 kDa) detected one d in culture (1) but not in the ganglion (T) as reported by Nicke *et al*. [56] C, relative optical density values of mature receptors at 1–4 days in culture (normalized with respect to β-tubulinIII) and compared to ganglion values. For all experiments shown in A-C n = 3 animals for ganglion or day in culture datapoint (each point is mean ± SEM). *: P < 0.05; **: P < 0.01.

We next assessed if rat and mouse TG neurons in culture demonstrated changes in the levels of mRNA transcripts of P2X and TRPV1 receptors with respect to the ganglion. To quantify changes in neuron specific mRNA neosynthesis for P2X and TRPV1 receptors in culture with respect to the ganglion, we performed real time RT-PCR experiments as a sensitive method to detect changes in gene expression [15]. Fig 2A shows that, when comparing samples containing an equal amount of the housekeeping gene GAPDH mRNA, there was significant upregulation of P2X_3 _and TRPV1 transcripts for rat and mouse neurons *in vitro *with respect to the ganglion after normalization for the neuronal-specific β-tubulinIII marker. In particular, P2X_3 _mRNA showed a peak at 24 h (1.29 fold, P < 0.05, and 1.39 fold, P < 0.01, for rat and mouse, respectively). Rat TRPV1 mRNA analysis showed a peak at 48 h (1.4 fold, P < 0.01), while in the mouse TRPV1 mRNA increased more substantially (1.9 fold, P < 0.001) already at the first d and remained elevated for the following two days. P2X_2 _mRNA signals did not show any significant change.

Because P2X_2 _and TRPV1 receptors are also expressed in non-neuronal cells, while the P2X_3 _receptor is not [16,17], it was important to assess the contribution of P2X_2_, P2X_3 _and TRPV1 mRNAs in 3 week-old cultures, containing no neurons (β-tubulinIII mRNA negative). Real time amplification experiments showed that equivalent amount of samples (measured as GAPDH mRNA amplification levels) gave undetectable amplification of P2X_2_, P2X_3 _and TRPV1 mRNAs, indicating that the contribution of non-neuronal mRNAs to PCR experiments was minimal.

Western immunoblotting experiments were performed to investigate protein expression levels for P2X_3_, P2X_2 _and TRPV1 receptors in rat and mouse ganglia as well in culture (Fig. 2B). To study the time course of the protein expression profiles in culture and in the ganglion, we investigated differences in the mature form of rat P2X_3_, P2X_2 _and TRPV1 receptors (bands at 57, 65, 120 kDa) under equal protein loading conditions assessed with β-tubulinIII staining (Fig. 2B, a and Fig. 2C). While in the case of the TRPV1 receptor there was a significant increment already at 24 h in culture (1.5 fold, P < 0.05), this was not paralleled by P2X_2 _and P2X_3 _expression that showed no substantial change in culture with respect to ganglion (Fig. 2C). Note that the 120 kDa TRPV1 protein was more prominent in culture than in the ganglion (Fig. 2B a). Moreover, in the case of the P2X_3 _receptor, intermediate bands (45–55 kDa) were observed in culture but not in the ganglia (Fig. 2B b), indicating that, despite a constant amount of the mature 57 kDa protein, there was a significant increment of P2X_3 _protein expression occurring in culture (2.0 fold, P < 0.05). Analysis of mouse 1–3 d cultures revealed similar protein expression behaviour (not shown). The different time profile of data related to TRPV1 expression obtained with real time RT-PCR and western blotting might have been due to a rapid increase in protein translation rate dependent on already present mRNA as reported for the same protein in DRG neurons *in vitro *[18]. Immunocytochemical analysis was then carried out to further investigate changes in receptor protein expression in culture.

### Immunoreactivity of TG neurons

**Figure 3:**
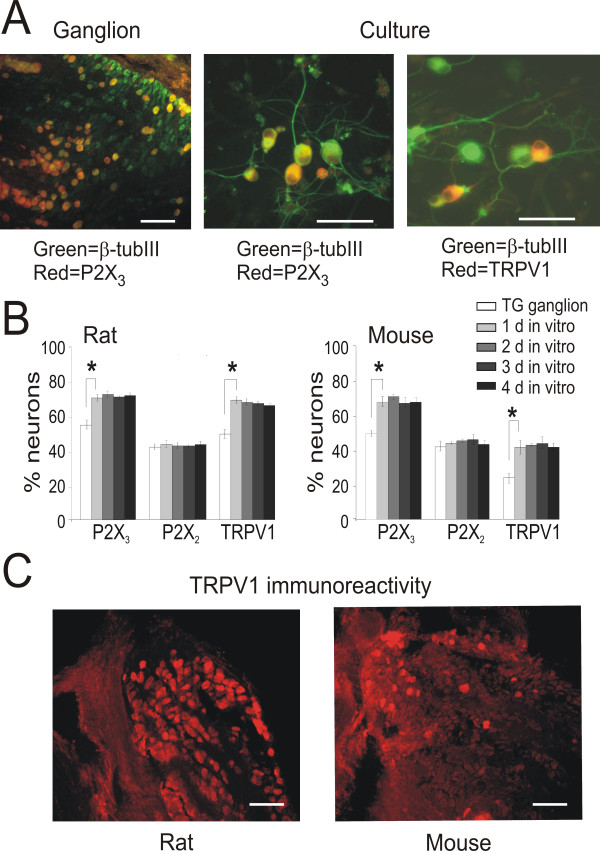
**P2X and TRPV1 immunoreactivity in ganglia and cultures**. A, example of P2X_3 _receptor expression by β-tubulinIII labeled neurons in a fixed ganglion (left; calibration bar = 50 μm). After one day in culture, medium-size neurons that are also labeled by β-tubulinIII are immunoreactive for P2X_3 _(middle) or TRPV1 (right; calibration bar = 50 μm) receptors. B, percentage of neurons (β-tubulinIII positive) immunoreactive for P2X_3_, P2X_2 _and TRPV1 in the ganglion and at different d in culture, for rat (left) and mouse (right; n = 3 animals in all cases). The number of P2X_3 _and TRPV1 immunoreactive neurons increase significantly after dissection and remain constant in culture, while P2X_2 _receptor labeled neurons remain unchanged. *: P < 0.05 for indicated pair of histograms. Values at later d in culture are also significantly different from ganglia. C, comparison of TRPV1 receptor immunoreactivity of rat (left) or mouse (right) ganglia. Note larger number of immunoreactive neurons in the rat tissue. Calibration bars = 50 μm.

The distribution among rat and mouse neurons of P2X_3_, P2X_2 _and TRPV1 receptors in freshly excised ganglia and TG cultures was studied as shown by the example of Fig. 3A in which rat ganglion immunoreactivity for P2X_3 _receptors is compared with 1 d cultured neurons. Neurons labeled by the P2X_3 _antibody were clearly detected, as much as neurons immunoreactive for TRPV1 receptors (right panel). Fig. 3B quantifies the percentage of TG neurons immunoreactive for P2X_3_, P2X_2 _and TRPV1 receptors under different experimental conditions. P2X_2 _receptors in both rat and mouse TGs were equally represented in ganglion and in culture (approximately 40 %). The number of rat and mouse P2X_3 _or TRPV1 immunoreactive neurons grew by about 20% with respect to the ganglion (see Fig. 3). Fig. 3C shows that the considerable difference between mouse and rat neurons expressing TRPV1 receptors in culture was not simply due to the *in vitro *condition: in fact, TRPV1 expressing neurons were clearly more abundant in rat ganglia than in mouse ones as quantified by comparing histograms in Fig. 3B.

Table 1 presents pooled data for the distribution of these receptors among rat and mouse neurons of different somatic diameter in the ganglion or in culture. P2X_3_, P2X_2 _and TRPV1 receptors were expressed by all three subpopulations of TG neurons (small, medium and large size cells) in the ganglion. It is noteworthy that the relative numbers of neurons expressing these receptors showed only minimal variations in culture (Table 1). Despite an increment in the P2X_3 _immunopositive cells in culture, P2X_3 _was predominantly confined to small size neurons for both rat and mouse (60%). In culture, P2X_2 _was expressed predominantly in medium (50%) while TRPV1 in small size neurons (65–70%). These values remained virtually unchanged during the time *in vitro*.

**Table 1:** P2X or TRPV1 of small, medium and large TG neurons in ganglia or in culture

**P2X**_3_						
	**Rat**			**Mouse**		

	**Small**	**Medium**	**Large**	**Small**	**Medium**	**Large**

**Tissue**	44 ± 4%	33 ± 3%	23 ± 4%	49 ± 3%	36 ± 4%	15 ± 4%
**24 h**	55 ± 3%	28 ± 3%	17 ± 2%	60 ± 4%	31 ± 3%	9 ± 2%
**48 h**	54 ± 2%	33 ± 3%	13 ± 3%	56 ± 4%	29 ± 3%	15 ± 2%
**72 h**	58 ± 5%	30 ± 4%	12 ± 2%	62 ± 5%	30 ± 4%	8 ± 2%
**96 h**	56 ± 4%	31 ± 4%	13 ± 3%	60 ± 4%	33 ± 3%	7 ± 2%

**P2X**_2_						

	**Rat**			**Mouse**		

	**Small**	**Medium**	**Large**	**Small**	**Medium**	**Large**

**Tissue**	30 ± 2%	55 ± 3%	15 ± 4%	32 ± 3%	52 ± 2%	16 ± 4%
**24 h**	34 ± 4%	56 ± 3%	10 ± 4%	36 ± 2%	51 ± 3%	13 ± 3%
**48 h**	36 ± 4%	57 ± 4%	7 ± 3%	29 ± 3%	55 ± 3%	16 ± 4%
**72 h**	33 ± 3%	57 ± 4%	10 ± 3%	22 ± 3%	65 ± 2%	13 ± 3%
**96 h**	34 ± 3%	60 ± 2%	6 ± 3%	21 ± 3%	69 ± 6%	10 ± 3%

**TRPV1**						

	**Rat**			**Mouse**		

	**Small**	**Medium**	**Large**	**Small**	**Medium**	**Large**

**Tissue**	60 ± 3%	31 ± 4%	9 ± 3%	59 ± 2%	32 ± 3%	9 ± 2%
**24 h**	65 ± 4%	29 ± 4%	6 ± 2%	66 ± 2%	31 ± 3%	3 ± 1%
**48 h**	64 ± 2%	29 ± 3%	7 ± 2%	70 ± 3%	28 ± 3%	2 ± 1%
**72 h**	66 ± 4%	29 ± 2%	5 ± 2%	68 ± 5%	30 ± 4%	2 ± 1%
**96 h**	68 ± 4%	27 ± 2%	5 ± 2%	72 ± 4%	26 ± 3%	2 ± 1%

Data are % distribution of P2X_3_, P2X_2 _or TRPV1 immunopositive neurons according to their somatic size (small: dia <15 μm; medium: 15 μm–25 μm; large: dia > 25 μm). In each box 100% = the total number of cells immunoreactive for the given receptor.

### Receptor co-expression

**Figure 4:**
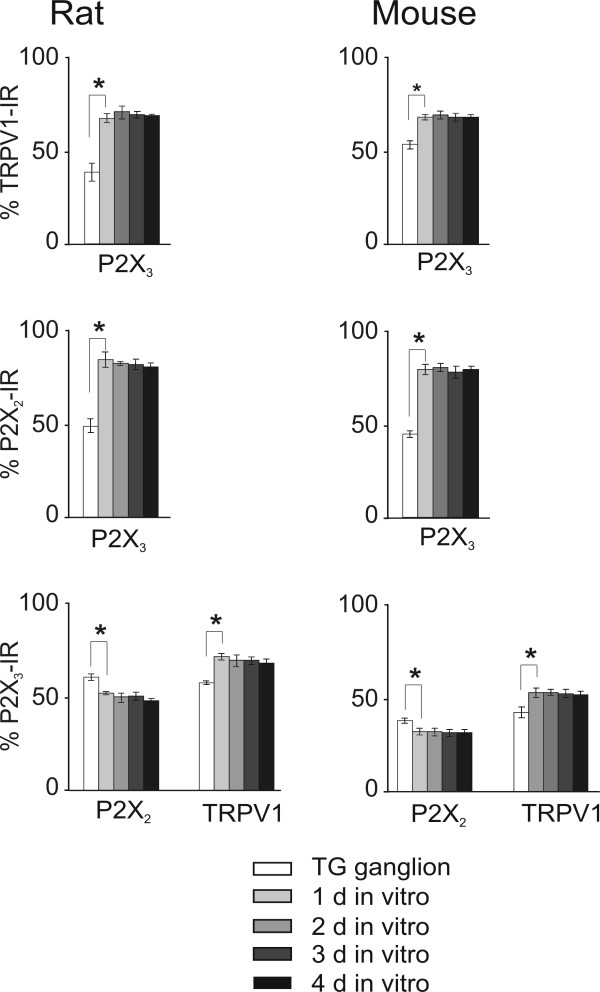
**Receptor co-localization investigated with double immunofluorescence**. Percent of neurons showing double immunoreactivity for pairs of P2X_3_, P2X_2 _and TRPV1 as indicated (rat data are on the left while mouse ones are on the right). The "reference" receptor type is taken as 100%. Data from ganglia (about 1,000 neurons) or at different d in culture (about 500 cells per culture) are shown with differently shaded histograms. n = 3 animals for each bar. *: P < 0.05. Values at later d in culture are also significantly different from ganglia. Further details are in the legend to Fig. 3.

We next studied the co-existence of two markers on same neurons. Pooled data are given in Fig. 4. Notwithstanding a large number of neurons co-expressing two markers, it was apparent that the number of P2X_3 _positive cells also positive for P2X_2 _receptors was higher in rat than in mouse cultures (53% vs 35%, respectively), a phenomenon observed also on ganglion tissue (63% vs 40%, respectively). Likewise, P2X_3 _positive cells had a higher probability of expressing TRPV1 receptors in rat than in mouse culture (75% vs 54%), a result confirmed with rat and mouse ganglia (61 and 43%, respectively). Although the global number of P2X_2 _receptor expressing neurons was always comparatively low (see Fig. 3 and Table 1), once in culture, the percentage of P2X_2_-positive neurons expressing also P2X_3 _receptors largely rose. A strong rise in the number of TRPV1-positive neurons that could also co-express P2X_3 _receptors occurred in rat TG culture and, less intensely, in mouse TG culture.

### Calcium imaging of TG neurons

To characterize the functional properties of a large number of intact (non-patched) neurons, we first performed intracellular Ca^2+^-imaging of 24 h cultured TG neurons activated by agonists of P2X_3 _and TRPV1 receptors as this method is a potent screening tool for P2X receptor assay [19]. As a pharmacological tool to differentiate between P2X_2 _and P2X_3 _receptors and to avoid activation of metabotropic P2Y receptors that are natively expressed by TG neurons [20], we used α, β-methylene-adenosine 5'-triphosphate (α, β-meATP), a selective agonist for P2X_3 _(and P2X_1_) subunit-containing receptors [16], and capsaicin to activate TRPV1 receptors [21].

**Figure 5:**
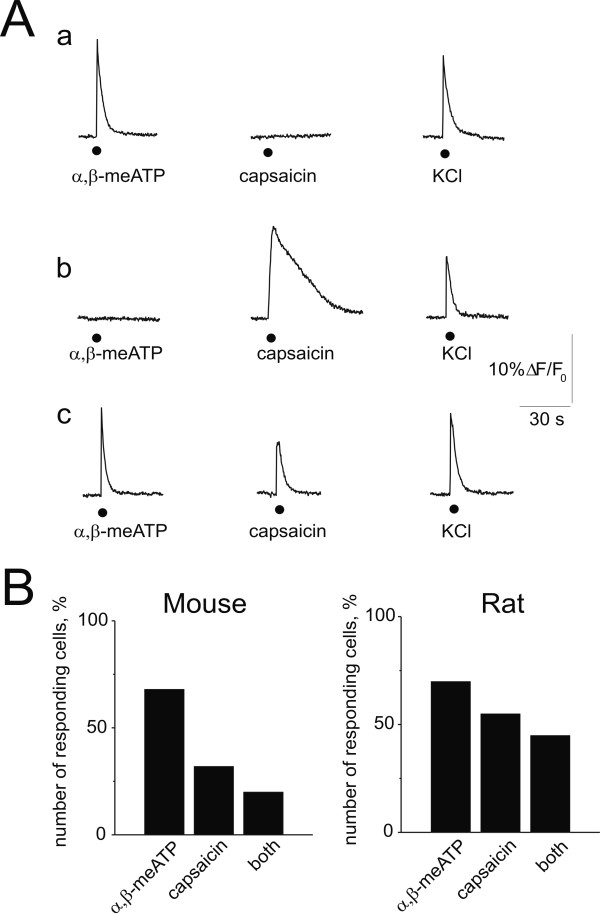
**Calcium imaging indicates functional expression of P2X and TRPV1 receptors**. A, examples of Ca^2+ ^transients in mouse TG neurons activated by 2 s application of 10 μM α, β-meATP or 1 μM capsaicin. a, example of cell responding to α, β-meATP only ('purinergic phenotype'); b, responding to capsaicin only ('vanilloid phenotype'); c, responding to both agonists ('mixed phenotype'). All cells respond to pulse application of KCl. B, number of neurons sensitive to α, β-meATP (regardless of their response to capsaicin), to capsaicin (regardless of their response to α, β-meATP) or to both agonists, for mouse (n = 346; left) and rat (n = 41; right) TG neurons. Neurons (kept in culture for 24 h) were identified by their responsiveness to 15 mM KCl (1 s), while satellite cells and fibroblasts did not respond to this agent. Note that the percent of cells responding to capsaicin was higher in the rat (55%) than in the mouse (33%).

Neurons were identified by their responsiveness to pulses of KCl (15 or 50 mM; 1 s). Fig. 5A shows examples of Ca^2+ ^transients in mouse TG neurons following 2 s application of 10 μM α, β-meATP or 1 μM capsaicin. Note that capsaicin-induced transients were often long lasting (see example in Fig. 5A b). KCl-sensitive cells could be classified into three groups: 1. responsive to α, β-meATP only ('purinergic phenotype'; Fig. 5A a); 2. responsive to capsaicin only ('vanilloid phenotype'; Fig. 5A b); 3. responsive to both agonists ('mixed phenotype'; Fig. 5A c). Figure 5B summarizes results obtained from 346 mouse and 41 rat TG neurons. In agreement with our immunocytochemistry results, most (68%) mouse neurons responded to α, β-meATP (regardless of their sensitivity to capsaicin), and 33% responded to capsaicin (regardless of their sensitivity to α, β-meATP). Twenty percent of mouse neurons responded to both drugs. Interestingly, the fraction of rat cells activated by α, β-meATP was almost the same (70%) as in the mouse, while the percentage of cells responding to capsaicin was higher (55%). The fraction of rat cells sensitive to both agonists was 44% (Fig. 5B). To sum up, imaging data from intact neurons validated the molecular biology and immunocytochemical profile of mouse and rat TG neurons.

### P2X or TRPV1 receptor-mediated currents generated by TG neurons in culture

Since in various tissues there are differences in P2X receptor function between rat and mouse cells [22,23], patch clamp recording was used to further characterize receptor-mediated currents of rat or mouse TG neurons (small and medium size).

**Figure 6:**
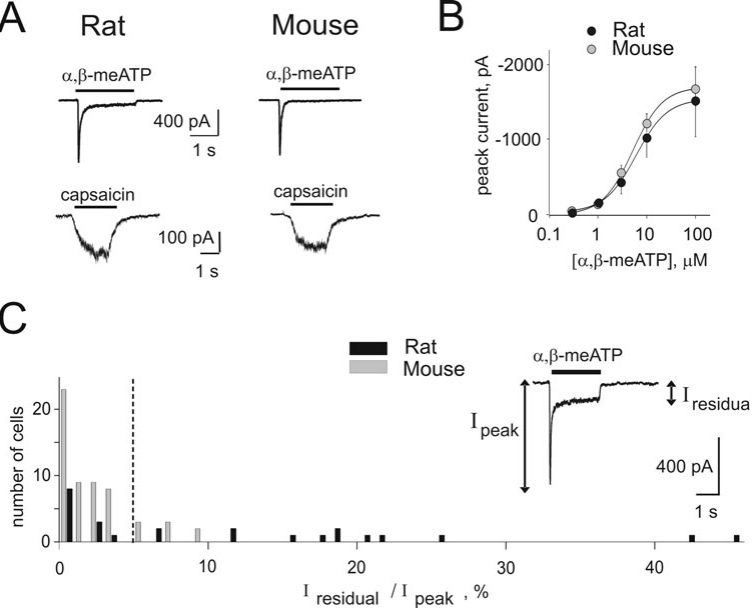
**Characteristics of pain receptors of cultured TG neurons**. A, examples of currents recorded from rat and mouse TG neurons in culture (24 h) during application of α, β-meATP (10 μM, 2 s; upper row) and capsaicin (10 μM, 2 s for rat; 1 μM, 2 s for mouse; bottom row). B, log dose-response curves for rat (filled symbols; n = 9) and mouse (grey symbols; n = 12) neurons cultured for 24 h. Both potency and efficacy of α, β-meATP were similar for rat and mouse. C, distribution of values of the residual current (I_residual_) present at the end of an application of α, β-meATP (10 μM, 2 s) in rat and mouse neurons cultured for 24 h, expressed as a fraction of the peak current (I_peak_; n = 24 and 58 for rat and mouse, respectively). Inset shows an example of the mixed type of current recorded from a subset of rat TG neurons. Dashed line indicates the 5% arbitrary threshold above which currents were considered to be mixed.

Fig. 6A shows typical examples of the currents induced by agonists of P2X_3_-containing or TRPV1 receptors. Application (2 s) of α, β-meATP (10 μM) to either rat or mouse neurons (top row) elicited a fast-developing inward current, which peaked and then strongly desensitized during agonist application, a behavior typical of currents mediated mainly by P2X_3 _receptors [16]. Plots of peak amplitude responses versus increasing concentrations of α, β-meATP are depicted in Figure 6B. For both species the plots attained analogous maximum response, and the EC_50 _values for α, β-meATP were almost the same in both species (5 ± 1 μM for rat neurons, n = 9, and 6 ± 1 μM for mouse neurons, n = 12). This observation is consistent with the realization that P2X_3 _receptors of rat and mouse share 98.7% identity of their primary sequence (Blast analysis: [NP_663501] and [NP_112337] for mouse and rat receptors, respectively).

Since one characteristic feature of P2X_3_-containing receptors is their fast-developing desensitization [16], the current decay during application of α, β-meATP (10 μM, 2 s) was also analyzed. The rapid phase of current decay could be fitted monoexponentially with a time constant (τ_fast_) which was the same for rat and mouse cells (44 ± 3 ms and 48 ± 3 ms for rat and mouse; n = 24 and 39, respectively), indicating that the onset of fast desensitization was the same for both species.

While α, β-meATP activates homomeric P2X_3 _receptors and has no effect on homomeric P2X_2 _receptors [16], this agonist can also activate heteromeric P2X_2/3 _receptors with characteristically-slow current decay during agonist application [24,25]. Thus, co-activation of P2X_3 _and P2X_2/3 _receptors would produce mixed responses with a fast peak and a residual current [24,25]. One index for the presence of heteromeric P2X_2/3 _receptors on TG neurons was considered the presence of a residual current (I_residual _in inset to Fig. 6C) at the end of a 2 s-long α, β-meATP application (10 μM). I_residual _was significantly (P < 0.01) different between rat and mouse neurons at 24 h in culture (-73 ± 22 pA, n = 27, and -15 ± 3 pA, n = 41 for rat and mouse cells, respectively). It was interesting to examine how many neurons would express responses indicative of heteromeric receptors. This issue is shown in Fig. 6C with the distribution of I_residual _(as % of the initial peak, I_peak_) for rat (filled bars) and mouse (grey bars) neurons. We assumed that currents comprising I_residual _at least 5 % of I_peak _were suggestive of heteromeric P2X_2/3 _receptors (vertical dashed line in Fig. 6C) because, under the present conditions, α, β-meATP was a selective agonist for P2X_3 _subunit-containing receptors. Using this criterion [26], 54 % of rat neurons generated mixed currents, while only 16 % of mouse neurons did so. These data suggested that α, β-meATP-mediated responses comprised a limited contribution by heteromeric P2X_2/3 _receptors. This notion was further supported by experiments with the selective P2X_3 _receptor antagonist A-317491 (1 μM; [27]) that almost completely abolished (5 ± 1 %; n = 13) the currents induced by α, β-meATP, confirming that P2X_3 _receptors were the target for the action of α, β-meATP.

Since approximately 40 % TG neurons were immunopositive for P2X_2 _subunits (Fig. 3B), we further explored the functional role of this subunit by comparing currents evoked by ATP with those induced by α, β-meATP (as the latter is ineffective on P2X_2 _receptors). On the same mouse TG neurons (24 h in culture), 10 μM ATP elicited currents with peak amplitude significantly larger (131 ± 9 %; P < 0.05) that the ones produced by α, β-meATP (n = 27). Interestingly, taking the criterion of 5 % size of I_residual_, while 3/27 neurons generated I_residual _in response to α, β-meATP, 10/27 produced a slow current to ATP application.

**Figure 7:**
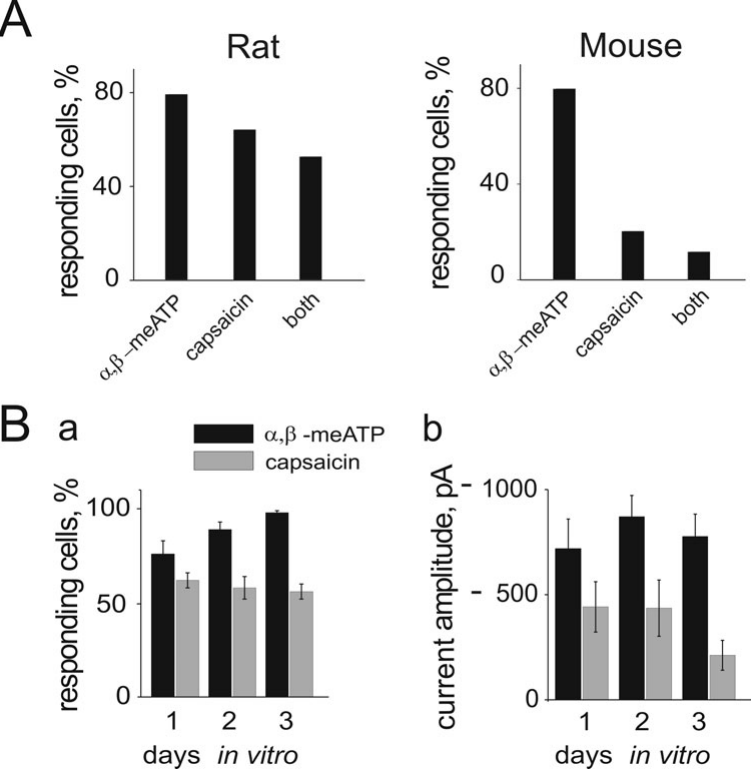
**Functional characterization of rat and mouse TG neurons**. A, fraction of cells responding to α, β-meATP (independently from their response to capsaicin), to capsaicin (independently from their response to α, β-meATP) or to both agonists for rat (left panel; n = 33) and mouse (right panel; n = 50). B, persistence of the responsiveness of rat TG neurons in culture to α, β-meATP and capsaicin. a, Proportion of cells responding to α, β-meATP (black bars; n = 7, 7, 10 cell cultures for the 1^st^, 2^nd ^and 3^rd ^day, respectively) and capsaicin (grey bars; n = 7, 8 and 8 cell cultures for the 1^st^, 2^nd ^and 3^rd ^day). b, peak amplitude of currents elicited by α, β-meATP (black bars; n = 22, 11 and 31 cells for the 1^st^, 2^nd ^and 3^rd ^day, respectively) and capsaicin (grey bars; n = 16, 13 and 10 cells for the 1^st^, 2^nd ^and 3^rd ^day). Only cells responding to the agonist were included in the current analysis.

Fig. 7A summarizes the proportion of rat and mouse cells (24 h in culture) responding to a test concentration of α, β-meATP or capsaicin. In the case of rat neurons, the number of cells sensitive to α, β-meATP was about 80 %, while 60 % responded to capsaicin. The percent value of cells responding to both agonists was about 50 %. In the case of mouse TG cells, approximately 80 % of neurons were activated by α, β-meATP, while about 20 % responded to capsaicin and only a small minority of neurons could respond to capsaicin as well as to α, β-meATP.

The next issue was the stability of functional receptors during culturing conditions. This was examined with rat TG neurons as shown in Fig. 7B a (filled bars) where the fraction of cells responding to α, β-meATP was large after 24 h in culture and only slightly increased later. The peak amplitude of their response, however, remained essentially unchanged (Fig. 7B b, filled bars). The number of cells responsive to capsaicin was, however, smaller and remained at a relatively steady level in culture (Fig. 7B a, grey bars), as the apparent decrease in response amplitude was not statistically significant (Fig. 7B b, grey bars).

### Chronically-applied NGF differentially modulated P2X_3 _and TRPV1 receptors

Although exogenous NGF is often used to grow sensory neurons in culture [4,12,28], we decided as a routine to avoid adding NGF to the medium because it is a potent algogenic substance and might thus alter the phenotype and function of pain receptors [1]. An ELISA bioassay was performed to measure the amount of NGF in the bulk medium of TG neurons after 24 h in culture. In rat and mouse culture medium NGF concentrations were 9.1 ± 1.3 and 12.5 ± 3.1 pg/ml (absolute concentrations were 6.4 ± 1.5 and 13.4 ± 1.7 pg, respectively): these values are not distant from the binding dissociation constant (0.6 nM) for the high affinity NGF receptor [29], especially when considering the large dilution in the bulk solution. Endogenous NGF was also detected in homogenates of ganglia or cells after 24 h in culture. Indeed, NGF was more concentrated in cultured cells (rat cultures = 19.5 ± 0.9 pg/μg of DNA content, n = 3 cultures; mouse cultures = 23.3 ± 2.2 pg/μg; n = 4) than in the whole ganglion (rat ganglia = 11.4 ± 1.1, n = 3 rats; mouse ganglia = 12.7 ± 0.1, n = 4).

**Figure 8:**
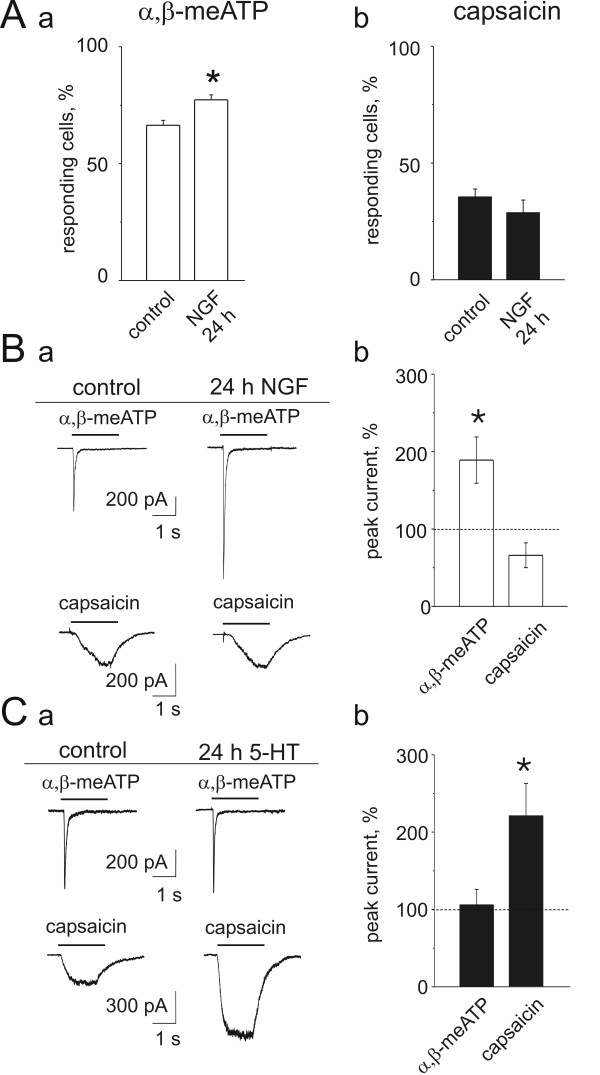
**Modulation of receptor function by chronically applied NGF or 5-HT**. A, Ca^2+ ^imaging of single neurons shows percent increase in mouse TG cells responsive to α, β-meATP (a) in control or after application of 50 ng/ml NGF (24 h; n = 6 culture dishes). In 6 sister cultures there was no significant change as far as responses to capsaicin (b) were concerned. *: P < 0.05. B, a, patch clamp current records show increased amplitude of mouse responses to α, β-meATP after 24 h NGF treatment, while responses to capsaicin remained equiamplitude. B, b, histograms summarizing the significant (*: P < 0.05) rise in α, β-meATP evoked current amplitude (n = 26) without significant change in capsaicin responses (n = 14). Data are expressed as % of control amplitude in sister cultures. C, 5-HT (10 μM; 24 h) upregulates the amplitude of rat capsaicin current without affecting responses to α, β-meATP (a). C,b shows significant rise in the peak current induced by capsaicin (n = 31) with no change in the α, β-meATP-evoked current (n = 31). Data are expressed as % of control amplitude in sister cultures. *: P < 0.05.

We next investigated what effects a dose of 50 ng/ml of NGF [12] applied for 24 h may produce on the function of P2X_3 _and TRPV1 receptors of mouse TG neurons. As a screening assay we first assessed changes in the percent of neurons responsive to α, β-meATP (10 μM) in terms of Ca^2+ ^transients: Fig. 8A a shows that, after NGF application, their number became significantly larger. Conversely, the fraction of neurons responsive to capsaicin (1 μM) remained unchanged (Fig. 8A b). These data were expanded with patch clamp investigations as indicated by the examples of Fig. 8B a that depicts larger membrane currents evoked by α, β-meATP (10 μM) and unchanged ones induced by capsaicin (1 μM) after NGF application. The histograms in Fig. 8B b summarize these data, indicating that chronically-applied NGF could selectively upregulate P2X_3 _receptor function with no significant change in the amplitude of capsaicin-induced currents. The lack of change in TRPV1 receptor mediated responses was not attributable to inability of the present cultures to show upregulation of capsaicin-induced responses by NGF. In fact, acutely applied NGF (50 ng/ml for 5 min) could upregulate responses (344 ± 158 %; n = 5; P < 0.05) induced by small concentrations of capsaicin (0.1 μM).

### Chronically-applied 5-HT selectively upregulated TRPV1 receptors

The differential upregulation of P2X_3 _and TRPV1 receptors in culture by NGF begged the question whether other mediators believed to be involved in headache might elicit similar long-lasting changes. Since short application of 5-HT has no action on P2X_3 _receptors [30], we investigated the effect of 5-HT (10 μM; [31]) applied for 24 h to rat TG neurons. Figure 8C a shows typical examples of α, β-meATP (10 μM)- or capsaicin (10 μM)- induced currents obtained in control or after treatment with 5-HT. Note that, while the α, β-meATP response was essentially the same, the response to capsaicin grew much larger. This phenomenon was statistically significant as indicated in Fig. 8C.

## Discussion

By combining morphology, molecular biology, immunocytochemistry, Ca^2+ ^imaging and electrophysiology, the current study provides the novel characterization of the expression, time-profile and function of P2X and TRPV1 receptors on rat or mouse TG neurons in culture. Comparing TG ganglia and cultures had not been done before and allowed us to discern distinctive changes in key pain transducing molecules in a species-dependent fashion and to monitor their early adaptive changes *in vitro*, thus demonstrating limits as well as usefulness of the model. Furthermore, the differential upregulation of P2X_3 _and TRPV1 receptors observed after chronically applying either NGF or 5-HT suggested diversity in adapting pain signaling systems to algogenic substances.

### Expression of P2X and TRPV1 receptors in ganglia and culture

Our culture conditions ensured that small and medium size neurons, normally associated with pain transducing function [21], were the largest population, in analogy with observations on sections of trigeminal ganglia. One important question was whether cultured TG neurons expressed P2X and TRPV1 receptors like those found in tissue sections of the TG. To the best of our knowledge, this issue has not been investigated before by directly comparing these markers in ganglion sections and in TG cultures.

Real time RT-PCR and Western blot analysis indicated that there was a significant increase in TRPV1 and P2X_3 _receptors in culture with little change for P2X_2 _receptors. In addition, the emergence of immature forms of P2X_3 _receptors may suggest neosynthesis and trafficking of these proteins to reflect ongoing plasticity. Although both RT-PCR and Western blot yielded unidirectional results, we observed a mismatch in the timecourse of RT-PCR and Western blot signals for TRPV1 receptors. A discrepancy between transcription and translation is not an uncommon phenomenon, as amply discussed in a recent review, because of the complex relationship between mRNA and protein as transcription and translation are governed by independent mechanisms and separate time constants [32].

While mouse ganglion P2X immunoreactivity data were comparable to those by Ruan *et al*.[33], immunostaining of cultured neurons showed that, in rat and mouse cultures, the number of cells containing P2X_3 _and TRPV1 subunits was significantly increased already at 24 h in culture and remained stable for the following 3 d. Increased immunoreactivity was, however, absent in the case of P2X_2 _receptors.

Co-expression of P2X and TRPV1 receptors was present in a large number of rat TG neurons, suggesting potential bimodal signaling to pain stimulants [2,21]. Interestingly, the small number of neurons immunoreactive for the P2X_2 _receptors acquired significant P2X_3 _receptor immunoreactivity in culture. This change in expression profile might provide the substrate for heteromeric assembly of P2X_2/3 _receptors, typically signaling chronic pain [2,5]. Overall, data from real time RT-PCR, Western blotting and immunocytochemistry suggest upregulation of P2X_3 _and TRPV1 receptors in culture with respect to the ganglion.

### Intracellular calcium imaging

Measuring responses only from excitable cells which generated Ca^2+ ^transients to a depolarizing pulse of KCl, we found the proportion of neurons responsive to α, β-meATP and the TRPV1 agonist capsaicin similar to the one obtained with immunocytochemistry data. Ca^2+ ^transients induced by α, β-meATP comprised Ca^2+ ^influx partly via open P2X_3 _receptors and mainly via depolarization activated Ca^2+ ^channels [34]. The complex nature of this Ca^2+ ^signal thus accounts for the slow decline of the α, β-meATP-evoked transients in comparison with the faster decay of membrane currents generated by the same agent (Fig. 6A). Our present imaging data thus show that functional receptors were expressed at membrane level on the majority of TG neurons.

### Membrane currents induced by α, β-meATP or capsaicin

Fast inward currents evoked by α, β-meATP were mediated by P2X_3 _receptors because of their block by the selective P2X_3 _blocker A-317491, and accord with knockout mouse model data indicating that sensory neurons almost exclusively express P2X_3 _and α, β-meATP-insensitive P2X_2 _receptors [35]. On a significant number of rat TG neurons, α, β-meATP produced mixed-type currents suggestive of co-expression of P2X_3 _and P2X_2/3 _receptors (the latter responsible for the residual current component). By using ATP it was possible to assess the functional role of P2X_2 _receptors. The amplitude of current responses evoked by ATP was somewhat larger and more frequently followed by a slow residual current, suggesting that P2X_2 _receptors demonstrated with molecular biology or immunocytochemistry could be functional, although their contribution to purinergic signaling was quite limited, in comparison to P2X_3 _receptors, in terms of response amplitude and percent of neurons expressing them.

Capsaicin-mediated responses were more rarely observed on mouse neurons than rat ones in accordance with the low expression level of TRPV1 receptors. Low TRPV1 immunoreactivity is reported also for other murine ganglia [36] while high expression is typical of the rat TG [37]. It is likely that, in the mouse, thermal nociception (which is normally mediated by TRPV1 receptors in the rat) is transduced, at least in part, by other nociceptive sensor proteins, in addition to TRPV1 receptors [36,38].

### NGF-induced modulation of P2X_3 _receptor function

Cultures of sensory neurons are often grown in the presence of high concentrations of NGF, known to be a strong algogen operating via multiple metabolic pathways [1,39]. Our study demonstrated that TG neurons could be grown in culture without exogenous NGF probably because the standard medium contained a small concentration of NGF synthesized by cultured cells as shown by the presence of NGF in homogenates of ganglia and cell cultures. Since the high-affinity binding of NGF to neuronal membranes has a 0.6 nM dissociation constant [29], it is probable that, in standard culture conditions, there was adequate production of NGF to preserve neuronal viability. It is likely that other endogenously-produced neurotrophins (like for example NT-3, BDNF and GDNF; [40-42] could also play a role in shaping the activity of these receptors.

Chronically-applied NGF upregulated the function of P2X_3 _receptors without changing TRPV1 receptor activity. This observation suggests a model system to test how an excess of NGF expected to occur during certain chronic pain states can bias the pain transducing properties of nociceptors towards purinergic rather than vanilloid function. Such effects by chronic NGF are distinct from the consequence of acute, brief application of this substance that enhanced TRPV1 receptor function as previously reported for DRG neurons [1,43].

### 5-HT-evoked modulation of TRPV1 receptors

While TG neurons express several types of 5-HT receptors [44] that can mediate acute nociception via rapid sensitization of TRPV1 receptors [9], much less is known about long-term consequences of 24 h exposure to this monoamine. This is an interesting issue because plasma levels (in the micromolar range) of 5-HT remain elevated for many hours during headache [45]. Chronic application of 5-HT to TG neurons in culture was followed by a large upregulation of TRPV1 receptor function without affecting P2X_3 _receptors. Because 5-HT induces a large, long-lasting increase in intracellular Ca^2+ ^[31], it is likely that Ca^2+ ^dependent changes in second messenger systems triggered such a plasticity of TRPV1 receptor function.

## Conclusion

Primary cultures of TG neurons could be maintained for a few days without the need of exogenous NGF. This approach provided a useful preparation to explore how chronically-applied algogens, implicated in headache pathophysiology, could generate heightened pain sensitivity. When NGF or 5-HT was tested on such a system, there was differential modulation of purinergic and vanilloid receptors, indicating distinct types of plasticity of nociceptors depending on the type of algogenic substance.

## Methods

### Cell culture preparation of TG neurons

Primary cultures of TG ganglion sensory neurons were obtained from C57-Black/6Jico mice or Wistar rats (P10–14; an age reported to show a quasi-adult phenotype for P2X receptors [33]). Animals were anesthetized by diethyl ether and decapitated (in accordance with the Italian Animal Welfare Act and approved by the Local Authority Veterinary Service). TG were rapidly excised and enzymatically dissociated in F12 medium (Invitrogen Corp, S.Giuliano Milanese, Italy) containing 0.25 mg/ml trypsin, 1 mg/ml collagenase and 0.2 mg/ml DNAse (Sigma) at 37°C. Cells were plated on poly-L-lysine-coated petri dishes in F12 medium with 10% fetal calf serum. For molecular biology or Ca^2+ ^imaging experiments, 2 × 10^6 ^cells were plated. For patch clamp experiments, mouse and rat cells were diluted twice. Three week-old TG cultures, that lacked neurons (i.e., negative for the neuron specific marker β-tubulinIII), served as negative control for molecular biology experiments.

### Real time RT-PCR

For PCR experiments, total RNA was extracted from TG ganglia or from culture using Trizol reagent (Invitrogen). After DNAse treatment (Ambion, Austin, TX, USA), cDNA synthesis and amplification were obtained using SuperScript III Two-step qRT-PCR kit (Invitrogen). Thirty ng cDNA were amplified with specific oligonucleotides and fluorogenic probes (TaqMan gene expression assays, Applied Biosystems, Applera, Norwalk, CT, USA) in ABI PRISM 7000 Sequence Detection System (Applied Biosystems) in the presence of ROX (Invitrogen) as internal reference dye. mRNA samples from fresh ganglia or cultures at different times were calibrated to obtain similar amplification of the GAPDH housekeeping mRNA. In preliminary experiments, analogous amplification of the samples was obtained also with 18S RNA probes. Nevertheless, to normalize the real time PCR results only with respect to neuronal mRNA, amplification of the neuronal specific β-tubulinIII housekeeping gene was chosen.

Specific TaqMan assays for mouse and rat target mRNA encoding P2X_3_, P2X_2 _and TRPV1, neuronal specific β-tubulinIII, GAPDH mRNA and 18S RNA (respective Applied Biosystems catalogue numbers: Mm00523699_m1, Mm00462952_m1, Mm01246282_m1, Rn01460299_m1, Mm00727586_s1, 4352339E FG, 4308329; www.appliedbiosystems.com) were chosen. All assays were validated for linearity of amplification efficiency and quantitative standard curves were obtained using serial dilutions of ganglia rat or mouse TG cDNA. To ensure absence of amplification artifacts, end point PCR products were initially assessed on ethidium bromide-stained agarose gels that gave a single band of the expected size for each assay. Negative controls containing no template cDNA were run in each condition and gave no results. The reactions were quantified when the PCR product of interest was first detected (cycle threshold). Calculations for relative mRNA transcript levels were performed using the comparative C_T _method (ΔΔC_T_) between cycle thresholds of different reactions [46]. In particular, the parameter C_T _(threshold cycle) is defined as the cycle number at which the fluorescence emission exceeds the fixed threshold. The calculation is based on the difference (ΔC_T_) between the C_T _values of the target receptor and the neuron-specific housekeeping gene (β-tubulinIII) at each time-point in culture, and then normalized with respect to the ΔC_T _value of the ganglion.

### Western immunoblot

Rat or mouse TG ganglia or cultures were homogenized in ice-cold lysis buffer containing 10 mM TrisHCl (pH 7.5), 150 mM NaCl, 20 mM EDTA, 1% Triton X-100, 8 M urea and protease inhibitors (Roche, Basel, Switzerland). The procedure was essentially the same as described by Fabbretti *et al*.[47]. The following polyclonal antibodies were used: P2X_3 _(1:2000 Neuromics, Bloomington, MN, USA), P2X_2 _or TRPV1 (1:400 Alomone, Jerusalem, Israel), β-tubulinIII (1:400, Chemicon, Temecula, CA, USA). To ensure correct equal loading reflecting the neuronal cell content in different lysates, protein extracts were quantified with bicinchonic acid (Sigma) and calibrated for the neuronal specific β-tubulinIII. The amount of loaded proteins was in the 20–50 μg/ml range.

### Immunocytochemistry

TG ganglion tissue was used with a free-floating immunostaining procedure. TG culture cells were fixed in 4% paraformaldehyde for 20 min at room temperature. The following rabbit polyclonal antibodies were used: P2X_3 _(1:500 from Chemicon), P2X_2 _and TRPV1 (1:200 from Alomone), and anti-cleaved caspase3 (1:100, Cell Signaling Technology, Beverly, MA, USA). The mouse monoclonal antibodies against the neuron specific β-tubulinIII (1:100, from Chemicon), GFAP (1:200, Sigma) and JNK (1:100, Santa Cruz) were used. Immunofluorescence reactions were visualized using the secondary antibodies AlexaFluor 488 or AlexaFluor 594 (1:500 dilution; Molecular Probes, Invitrogen). The P2X_3 _antibody used for double immunofluorescence experiments was obtained by immunizing a guinea-pig with the peptide CVEKQSTDSGAYSIGH. The specificity of the guinea-pig anti-P2X_3 _antibody was evaluated by western immunoblotting and immunofluorescence experiments (dilution 1:500 and 1:200, respectively) of HEK-293 cells transfected with pCDNA-P2X_3 _[47]. Tissue or cells stained with the secondary antibody only showed no immunostaining. To minimize tissue autofluorescence, TG ganglia were treated with Sudan Black. All images were captured under the same brightness and contrast settings. Control experiments using pre-immune guinea-pig serum gave no signal. In each experiment the number of positive neurons for a given antibody was normalized by dividing the number of positive cells by the number of β-tubulinIII-stained cells (equal to 100 %). For double immunofluorescence experiments the number of neurons stained with a certain antibody was referred as a percent of the total number of cells stained with the other antibody. An average of 500 cells in culture or 1,000 cells in the tissue were counted for each condition. Each data is the mean of at least 3 independent experiments. Results were analyzed with the ImagePro Express software (Media Cybernetics, L.P., Silver Spring, MD, USA).

### Scanning electron microscopy

For scanning electron microscopy, 24 h rat or mouse TG cultures were fixed in 2.5% glutaraldehyde (Sigma; in 0.1 buffered phosphate; pH 7.3) for 30 min at 4°C and postfixed with 1% OsO_4 _(Sigma), dehydrated in ethanol and dried by the critical-point method [44]. Ganglia or cultures were sputter-coated with gold (Electron microscopy sciences, Hatfield, PA, USA) as described [48]. Specimens were observed under a Stereoscan 430i microscope (Leica, Houston, TX, USA). Three hundred cells obtained from 4 different rats and 6 mice were observed. Freshly dissected TGs from two rats were slit open after de-sheathing without enzymatic treatment. Ganglia were fixed for 3 h at 4°C and treated as above.

### ELISA

The level of NGF present in the supernatant or in the cell lysates of TG culture was assessed with using the Emax NGF immunoassay system (Promega, Madison, WI, USA). Rat and mouse TG culture medium was collected after 24 h from plating and concentrated 4-fold for analysis (n = 4). Furthermore, mouse or rat ganglia or cell cultures were homogenated in 200 μl of a buffer containing 137 mM NaCl, 20 mM TrisHCl (pH 8), 1% NP40, 10% glycerol and protease inhibitors (Roche). Samples were diluted (1:6) in the same buffer and processed for ELISA assay. Results were corrected for blank and normalized. In the case of ganglia or cells, quantification was normalized with respect to the genomic DNA content (purified using GenElute mammalian genomic DNA kit, Sigma). The recovery of exogenously added NGF (100 ng/ml) was 63 ± 11 % (n = 3); data were not corrected for recovery. No NGF was detected in the fetal calf serum before adding it to the cultures.

### Patch-clamp recording

Cells were continuously superfused (2 ml/min) with physiological solution containing (in mM): 152 NaCl, 5 KCl, 1 MgCl_2_, 2 CaCl_2_, 10 glucose and 10 HEPES (pH adjusted to 7.4 with NaOH, osmolarity adjusted to 320 mOsm with glucose). Single cells were patch clamped in the whole-cell configuration using pipettes with a resistance of 3–4 MΩ when filled (in mM) with 140 KCl, 0.5 CaCl_2_, 2 MgCl_2_, 2 Mg_2_ATP_3_, 2 GTP, 10 HEPES and 10 EGTA (pH adjusted to 7.2 with KOH; osmolarity 285 mOsm). Currents were recorded from medium-sized (15–25 μm) nociceptive TG neurons [1]. TG cells were voltage clamped at membrane potential ranging from -70 to -60 mV. Series resistance was compensated by 70 %. α, β-meATP concentration-response curves were obtained by applying the same dose range to each tested cell; results were fitted with a sigmoidal curve (Origin 6.0, Microcal, Northampton, MA, USA) in order to express agonist potency in terms of EC_50 _values (concentration producing 50 % of the maximum response). Each concentration of α, β-meATP was applied for 2 s every 7 min to obtain full response recovery from desensitization. Capsaicin induces responses with strong tachyphylaxis during repetitive applications [49] and it can kill primary afferent nociceptors [50]: to circumvent these problems, a single dose of capsaicin was applied to each TG neuron. On mouse neurons, 1 μM capsaicin was used as standard test dose to yield reproducible inward currents, because even a small increment in concentration (10 μM) produced very slowly reversible inward currents as previously reported for sensory neurons [51]. On rat neurons, reproducible responses were evoked with a test (1–10 μM) concentration of capsaicin. This concentration is in excess of the EC_50 _value for TG neurons [52]. In order to minimize any possible difference in responses between TG neuron preparations, sister dishes were used on each occasion to compare control neurons and neurons treated with 5-HT or NGF (acute or chronic treatment).

### Calcium imaging

Cells were incubated for 40 min at 20–22°C in physiological solution containing Fluo3 (AM ester cell-permeable compound; 5 μM; Molecular Probes), followed by a 30 min washout period. Fluorescence emission was detected with a fast CCD camera (Coolsnap HQ; Roper Scientific, Duluth, GA, USA). Images were acquired with 150 ms exposure time and single cell responses were analysed with the Metafluor software (Metafluor Imaging Series 6.0, Universal Imaging Corporation, Downingtown, PA, USA). Intracellular Ca^2+ ^transients were expressed as percent amplitude increase (ΔF/F_0_, where F_0 _is the baseline fluorescence level and ΔF is the increment over baseline). Each event was also visually inspected to exclude artifactual components.

### Drug delivery in functional experiments

α, β-meATP, NGF and capsaicin (all from Sigma) were diluted with physiological solution to final concentration and applied by a rapid superfusion system (Rapid Solution Changer RSC-200, BioLogic Science Instruments, Grenoble, France). The time for solution exchange was about 30 ms [34]). Chronic application of NGF (50 ng/ml) was done by applying this substance to TG cultures for 24 h; cells were patch clamped immediately after washing out this dose of NGF. Twenty-four hour long treatment with 5-HT (10 μM) was carried out in the continuous presence of the monoaminoxidase inhibitor pargyline (100 μM; Sigma) to prevent enzymatic breakdown of this monoamine. Parallel controls were treated with the same concentration of pargyline alone.

### Data analysis

Data are presented as the means ± standard error of the mean (n = number of cells, unless otherwise indicated). The statistical significance was assessed with Mann-Whitney rank-sum test and the Wilcoxon test for non parametric data, and with Student's *t*-test for parametric data (KyPlot, version 2.0, Qualest Co., www.qualest.co.jp). For RT-PCR and Western blot, the films were scanned and band density was measured using CorelDraw Photopaint software (Corel, Berkshire, UK), normalized with β-tubulinIII control band and compared to the tissue value. For real time PCR, the relative mRNA expression of P2X_3_, P2X_2 _and TRPV1 in the different samples was normalized to the neuronal β-tubulinIII mRNA content in each condition and correlated with the one of the TG tissue. These experiments were performed in duplicate and repeated thrice for mouse and rat samples. Differences between groups were compared using ANOVA. A P value of < 0.05 was accepted as indicative of a statistically significant difference.

## Abbreviations

α, β-meATP: α, β-methyleneadenosine 5'-triphosphate. DRG: dorsal root ganglion. EC_50_: concentration producing 50 % of the maximum response. NGF: nerve growth factor. P2X: membrane receptors gated by ATP. TG: trigeminal ganglion. TRPV1: membrane channel gated by capsaicin. 5-HT: serotonin.

## Competing interests

The author(s) declare that they have no competing interests.

## Authors' contributions

MS, MD and EF performed the molecular biology and immunocytochemical studies. MZ performed the electron microscopy. AF and RG performed electrophysiology and imaging. AN oversaw the research. The manuscript was jointly prepared by all authors.

## References

[B1] Julius D, Basbaum AI (2001). Molecular mechanisms of nociception. Nature.

[B2] North RA (2003). The P2X_3 _subunit: a molecular target in pain therapeutics. Curr Opin Investig Drugs.

[B3] Cook SP, Vulchanova L, Hargreaves KM, Elde R, McCleskey EW (1997). Distinct ATP receptors on pain-sensing and stretch-sensing neurons. Nature.

[B4] Spehr J, Spehr M, Hatt H, Wetzel CH (2004). Subunit-specific P2X-receptor expression defines chemosensory properties of trigeminal neurons. Eur J Neurosci.

[B5] Jarvis MF (2003). Contributions of P2X_3 _homomeric and heteromeric channels to acute and chronic pain. Expert Opin Ther Targets.

[B6] Durham PL, Russo AF (2003). Stimulation of the calcitonin gene-related peptide enhancer by mitogen-activated protein kinases and repression by an antimigraine drug in trigeminal ganglia neurons. J Neurosci.

[B7] Hu WP, Guan BC, Ru LQ, Chen JG, Li ZW (2004). Potentiation of 5-HT_3 _receptor function by the activation of coexistent 5-HT receptors in trigeminal ganglion neurons of rats. Neuropharmacology.

[B8] Goadsby PJ, Lipton RB, Ferrari MD (2002). Migraine–current understanding and treatment. N Engl J Med.

[B9] Sugiuar T, Bielefeldt K, Gebhart GF (2004). TRPV1 function in mouse colon sensory neurons is enhanced by metabotropic 5-hydroxytryptamine receptor activation. J Neurosci.

[B10] Sarchielli P, Alberti A, Floridi A, Gallai V (2001). Levels of nerve growth factor in cerebrospinal fluid of chronic daily headache patients. Neurology.

[B11] Price TJ, Louria MD, Candelario-Soto D, Dussor GO, Jeske NA, Patwardhan AM, Diogenes A, Trott AA, Hargreaves KM, Flores CM (2005). Treatment of trigeminal ganglion neurons in vitro with NGF, GDNF or BDNF:effects on neuronal survival, neurochemical properties and TRPV1-mediated neuropeptide secretion. BMC Neurosci.

[B12] Cook SP, McCleskey EW (1997). Desensitization, recovery and Ca^2+^-dependent modulation of ATP-gated P2X receptors in nociceptors. Neuropharmacology.

[B13] Ambalavanar R, Moritani M, Dessem D (2005). Trigeminal P2X_3 _receptor expression differs from dorsal root ganglion and is modulated by deep tissue inflammation. Pain.

[B14] Matsuda S, Desaki J, Aburaja J, Sakanaka M (1997). Perikarial projections of developing spinal ganglion neurons in the chick demonstrated by scanning electron microscopy. Anal Embriol.

[B15] Huggett J, Dheda K, Bustin S, Zumla A (2005). Real-time RT-PCR normalisation; strategies and considerations. Genes Immun.

[B16] North RA (2002). Molecular physiology of P2X receptors. Physiol Rev.

[B17] Toth A, Boczan J, Kedei N, Lizanecz E, Bagi Z, Papp Z, Edes I, Csiba L, Blumberg PM (2005). Expression and distribution of vanilloid receptor 1 (TRPV1) in the adult rat brain. Mol Brain Res.

[B18] Ji RR, Samad TA, Jin SX, Schmoll R, Woolf CJ (2002). p38 MAPK activation by NGF in primary sensory neurons after inflammation increases TRPV1 levels and maintains heat hyperalgesia. Neuron.

[B19] He ML, Zemkova H, Koshimizu TA, Tomic M, Stojilkovic SS (2003). Intracellular calcium measurements as a method in studies on activity of purinergic P2X receptor channels. Am J Physiol Cell Physiol.

[B20] Ruan HZ, Burnstock G (2003). Localisation of P2Y_1 _and P2Y_4 _receptors in dorsal root, nodose and trigeminal ganglia of the rat. Histochem Cell Biol.

[B21] Caterina MJ, Julius D (2001). The vanilloid receptor: a molecular gateway to the pain pathway. Annu Rev Neurosci.

[B22] Hibell AD, Kidd EJ, Chessell IP, Humphrey PP, Michel AD (2000). Apparent species differences in the kinetic properties of P2X_7 _receptors. Br J Pharmacol.

[B23] Ma B, Ruan HZ, Cockayne DA, Ford AP, Burnstock G, Dunn PM (2004). Identification of P2X receptors in cultured mouse and rat parasympathetic otic ganglion neurones including P2X knockout studies. Neuropharmacology.

[B24] Lewis C, Neidhart S, Holy C, North RA, Buell G, Surprenant A (1995). Coexpression of P2X_2 _and P2X_3 _receptor subunits can account for ATP-gated currents in sensory neurons. Nature.

[B25] Burgard EC, Niforatos W, van BiesenT, Lynch KJ, Touma E, Metzger RE, Kowaluk EA, Jarvis MF (1999). P2X receptor-mediated ionic currents in dorsal root ganglion neurons. J Neurophysiol.

[B26] Grubb BD, Evans RJ (1999). Characterization of cultured dorsal root ganglion neuron P2X receptors. Eur J Neurosci.

[B27] Jarvis MF, Burgard EC, McGaraughty S, Honore P, Lynch K, Brennan TJ, Subieta A, Van BiesenT, Cartmell J, Bianchi B, Niforatos W, Kage K, Yu H, Mikusa J, Wismer CT, Zhu CZ, Chu K, Lee CH, Stewart AO, Polakowski J, Cox BF, Kowaluk E, Williams M, Sullivan J, Faltynek C (2002). A-317491, a novel potent and selective non-nucleotide antagonist of P2X_3 _and P2X_2/3 _receptors, reduces chronic inflammatory and neuropathic pain in the rat. Proc Natl Acad Sc.

[B28] Viana F, de la Pena E, Pecson B, Schmidt RF, Belmonte C (2001). Swelling-activated calcium signalling in cultured mouse primary sensory neurons. Eur J Neurosci.

[B29] Hartman DS, McCormack M, Schubenel R, Hertel C (1992). Multiple trkA proteins in PC12 cells bind NGF with a slow association rate. J Biol Chem.

[B30] Nakazawa K, Ohno Y (1997). Effects of neuroamines and divalent cations on cloned and mutated ATP-gated channels. Eur J Pharmacol.

[B31] Durham PL, Russo AF (1999). Regulation of calcitonin gene-related peptide secretion by a serotonergic antimigraine drug. J Neurosci.

[B32] Lewandowski NM, Small SA (2005). Brain microarray: finding needles in molecular haystacks. J Neurosci.

[B33] Ruan HZ, Moules E, Burnstock G (2004). Changes in P2X_3 _purinoceptors in sensory ganglia of the mouse during embryonic and postnatal development. Histochem Cell.

[B34] Sokolova E, Nistri A, Giniatullin R (2001). Negative cross talk between anionic GABA_A _and cationic P2X ionotropic receptors of rat dorsal root ganglion neurons. J Neurosci.

[B35] Cockayne DA, Dunn PM, Zhong Y, Rong W, Hamilton SG, Knight GE, Ruan HZ, Ma B, Yip P, Nunn P, McMahon SB, Burnstock G, Ford AP (2005). P2X_2 _knockout mice and P2X_2_/P2X_3 _double knockout mice reveal a role for the P2X_2 _receptor subunit in mediating multiple sensory effects of ATP. J Physiol.

[B36] Caterina MJ, Leffler A, Malmberg AB, Martin WJ, Trafton J, Petersen-Zeitz KR, Koltzenburg M, Basbaum AI, Julius D (2000). Impaired nociception and pain sensation in mice lacking the capsaicin receptor. Science.

[B37] Ichikawa H, Sugimoto T (2001). VR1-immunoreactive primary sensory neurons in the rat trigeminal ganglion. Brain Res.

[B38] Woodbury CJ, Zwick M, Wang S, Lawson JJ, Caterina MJ, Koltzenburg M, Albers KM, Koerber HR, Davis BM (2004). Nociceptors lacking TRPV1 and TRPV2 have normal heat responses. J Neurosci.

[B39] Andreev NY, Dimitrieva N, Koltzenburg M, McMahon SB (1995). Peripheral administration of nerve growth factor in the adult rat produces a thermal hyperalgesia that requires the presence of sympathetic post-ganglionic neurons. Pain.

[B40] Sariola H, Saarma M (2003). Novel functions and signalling pathways for GDNF. J Cell Sci.

[B41] Price TJ, Louria MD, Candelario-Soto D, Dussor GO, Jeske NA, Patwardhan AM, Diogenes A, Trott AA, Hargreaves KM, Flores CM (2005). Treatment of trigeminal ganglion neurons in vitro with NGF, GDNF or BDNF: effects on neuronal survival, neurochemical properties and TRPV1-mediated neuropeptide secretion. BMC Neurosci.

[B42] Wilson-Gerwing TD, Dmyterko MV, Zochodne DW, Johnston JM, Verge VM (2005). Neurotrophin-3 suppresses thermal hyperalgesia associated with neuropathic pain and attenuates transient receptor potential vanilloid receptor-1 expression in adult sensory neurons. J Neurosci.

[B43] Bonnington JK, McNaughton PA (2003). Signalling pathways involved in the sensitisation of mouse nociceptive neurons by nerve growth factor. J Physiol.

[B44] Lazarov NE (2002). Comparative analysis of the chemical neuroanatomy of the mammalian trigeminal ganglion and mesencephalic trigeminal nucleus. Prog Neurobiol.

[B45] Ferrari MD, Odink J, Tapparelli C, Van KempenGM, Pennings EJ, Bruyn GW (1989). Serotonin metabolism in migraine. Neurology.

[B46] Livak KJ, Schmittgen TD (2001). Analysis of relative gene expression data using real-time quantitative PCR and the 2^-ΔΔ*C*^_T _method. Methods.

[B47] Fabbretti E, Sokolova E, Masten L, D'Arco M, Fabbro A, Nistri A, Giniatullin R (2004). Identification of negative residues in the P2X_3 _ATP receptor ectodomain as structural determinants for desensitization and the Ca^2+^-sensing modulatory sites. J Biol Chem.

[B48] Robards AW, Wilson AJ (1993). Procedures in Electron Microscopy.

[B49] Koplas PA, Rosenberg RL, Oxford GS (1997). The role of calcium in the desensitization of capsaicin responses in rat dorsal root ganglion neurons. J Neurosci.

[B50] Caterina MJ, Schumacher MA, Tominaga M, Rosen TA, Levine JD, Julius D (1997). The capsaicin receptor: a heat-activated ion channel in the pain pathway. Nature.

[B51] Kirschstein T, Greffrath W, Busselberg D, Treede RD (1999). Inhibition of rapid heat responses in nociceptive primary sensory neurons of rats by vanilloid receptor antagonists. J Neurophysiol.

[B52] Liu L, Lo Y, Chen I, Simon SA (1997). The responses of rat trigeminal ganglion neurons to capsaicin and two nonpungent vanilloid receptor agonists, olvanil and glyceryl nonamide. J Neurosci.

[B53] Vulchanova L, Riedl MS, Shuster SJ, Buell G, Surprenant A, North RA, Elde R (1997). Immunohistochemical study of the P2X_2 _and P2X_3 _receptor subunits in rat and monkey sensory neurons and their central terminals. Neuropharmacology.

[B54] Newbolt A, Stoop R, Virginio C, Surprenant A, North RA, Buell G, Rassendren F (1998). Membrane topology of an ATP-gated ion channel (P2X receptor). J Biol Chem.

[B55] Kedei N, Szabo T, Lile JD, Treanor JJ, Olah Z, Iadarola MJ, Blumberg PM (2001). Analysis of the native quaternary structure of vanilloid receptor 1. J Biol Chem.

[B56] Nicke A, Baumert HG, Rettinger J, Eichele A, Lambrecht G, Mutschler E, Schmalzing G (1998). P2X_1 _and P2X_3 _receptors form stable trimers: a novel structural motif of ligand-gated ion channels. EMBO J.

